# Miscellany

**Published:** 1894-08

**Authors:** 


### Miscellany.

---

Laxol is a new preparation of castor oil that is absolutely palatable, as will be seen by reference to advertising page xiii., where it appears for the first time.

---

Frederick P. Stearns & Co., of Detroit, are among the new advertisers in this issue, and their card will be found on page xxiv. of the advertising forms.

Mortality of Tuberculosis.—Dr. Lagneau said that his investigation into the relationship existing between occupations and the development of tuberculosis showed that the greatest number of deaths from phthisis occurred in workers exposed to irritating substances in the respired air. In Switzerland, ten out of 100 stone cutters die from phthisis. In England, 1,000 deaths occurring in these workers, 340 were from phthisis. Tuberculosis makes cruel onslaught likewise in those individuals who habitually occupy a bent posture at their occupations, and in those who live sedêntary and intellectual lives. Of 1,000 deaths in Italy among students and seminarians, 450 died of phthisis—that is, nearly one-half. In England, of a similar number of deaths in printers, 430 died of phthisis.

On the other hand, statistics show that it is quite exceptional for this disease to be the cause of death of those who live in open air. In Switzerland, of 1,000 deaths occurring in outdoor laborers and farmers, not more than one or two die from phthisis. A similar number of deaths in Italy among shepherds and farmers shows only from forty-four to fifty-five deaths.

In France the sanitary statistics gathered from 662 towns show that the more the population is conglomerated, so in proportion are the inhabitants gravely infected with tuberculosis.—*Medical Record.*

———

The Association of American Medical Colleges.—The following resolutions were adopted at a meeting held in San Francisco, Cal., June 7, 1894:

*Resolved*, That colleges, members of this association, shall require of all matriculates an examination as follows:

1. An English composition in the handwriting of the applicant of not more than 200 words; said composition to include construction, punctuation and spelling.

2. Arithmetic, fundamental rules, common and decimal fractions and ratio and proportion.

3. Algebra—through quadratics.

4. Physics—elementary—Gage.

5. Latin—an amount equal to one year's study, as indicated in Harkness' Latin reader.

(The above resolution does not apply to students exempt from the entrance examination, as per Sec. 2, Art. III.)

*Resolved*, That the following classes of students be recognized as entitled to apply for advanced standing in colleges members of this body.

*a.* Such graduates of recognized colleges and universities as have completed the prescribed courses in chemistry and biology therein.

*b.* Graduates and matriculates of colleges of homeopathy.

*c.* Graduates and matriculates of colleges of eclectic medicine.

*d.* Graduates and matriculates of colleges of dentistry requiring two or more courses of lectures before conferring the degree of D. D. S.

*e.* Graduates and matriculates of colleges of pharmacy.

*f.* Graduates and matriculates of colleges of veterinary medicine.

It is proved, however, that the above class of students be required to comply with the provisions of the entrance examination and to prove their fitness to advance standing by an individual examination upon each branch below the class he or she may desire to enter.

*Resolved*, That students graduating in 1889 or subsequent classes be required to pursue the study of medicine four years and to have attended four annual courses of lectures of not less than six months' duration each.

---

THE MR. TAIT FUND.—On July 5th the subscribers to this fund met at the residence of Dr. Glover, Highbury, the chairman of the committee. The meeting was representative, and letters were read from Sir Dyce Duckworth, Dr. John Williams, Mr. Tom Smith, Mr. Marsh, Dr. Brunton, and others, expressing regret at being unable to attend. Dr. Glover, in the name of the subscribers, presented Dr. Tait with a cheque for £162 19s. 3d., which with £2 19s. 9d. expenses for circulars, postage, etc., made £165 19s. 0d., the sum raised. This represents about five-sixths of Dr. Tait's legal expenses. The chairman, while enjoining on all practitioners the duty of becoming members of defense unions, expressed the hope that if such actions should still occur they might always have in the defendant the advantage they had in this case—one whose history and whose character were a sufficient reply to all charges. The interest of the occasion was completed by the presentation, on behalf of Dr. Tait's patients, of a very handsomely illuminated address, expressing unabated respect for Dr. Tait, and signed by about 200 of his *clientéle.* The address was presented by Mr. Wm. Muller, of Highbury New Park. Dr. Trait made a very graceful and suitable reply. . . . .—*British Med. Jour.*, July 14, 1894.

